# A hospital-based observational study on HIV-TB co-infection

**DOI:** 10.1099/acmi.0.000787.v4

**Published:** 2025-08-06

**Authors:** Akansha Soni, Vimala Venkatesh, Parul Jain, Amita Jain, D. Himanshu Reddy, Neetu Gupta, Ritu Tandon

**Affiliations:** 1 Department of Microbiology, King George's Medical University, Lucknow, India; 2 Department of Medicine, King George’s Medical University, Lucknow, India; 3 A.R.T Center, King George’s Medical University, Lucknow, India

**Keywords:** anti retroviral therapy, anti-tubercular treatment, CD4 counts, co-infection, human immunodeficiency virus (HIV), tuberculosis

## Abstract

**Background.** Human immunodeficiency virus (HIV) is the major cause of failure to reach targets of tuberculosis (TB) control in settings with high HIV loads. TB, on the other hand, enhances the progression of HIV infection to AIDS. This study was done to understand the epidemiological and clinical profile of HIV-TB co-infected patients and to study the impact of TB on the recovery of CD4 counts.

**Methodology.** An observational study was conducted in which of the 573 patients newly diagnosed with HIV infection and enrolled at the antiretroviral therapy (ART) centre, King George’s Medical University, Lucknow, between May 2021 and June 2022, 80 patients who also had newly diagnosed TB were included. These HIV-TB co-infected patients were analysed for demographic factors. Also, clusters of differentiation 4 (CD4) counts were done at the time of enrolment on ART and then later, ~6 to 8 months of recieving ART and anti-tubercular treatment (ATT) initiation. For comparison, of the 493 HIV-only patients, 50 age- and gender-matched consecutive patients for whom baseline and follow-up CD4 counts were available were enrolled as controls. The change from baseline CD4 count was calculated using a paired t-test and Wilcoxon signed rank test.

**Results.** In the present study, among HIV-TB co-infected patients, baseline CD4 levels were 194.52±162.27, and follow-up CD4 levels were 285.09±170.33. A statistically significant increment of 90.57±165.60 in mean CD4 levels was observed (*t*=4.019; *P*<0.001). Likewise, in only HIV-positive patients, a statistically significant increment of 125.26±191.48 (35.75%) cells in mean CD4 levels was observed (*t*=4.626; *P*<0.001). The increase in CD4 counts in HIV only population was significantly higher than that observed in HIV-TB co0infected patients.

**Conclusion.** Though significant rise in CD4 counts was observed in both HIV-TB co-infected patients and HIV-only patients after 6 to 8 months of appropriate therapy, the rise was significantly higher among the HIV-only group as compared to the HIV-TB co-infected group.

## Data Summary

For this manuscript, no supporting or new data, tools, software or code were generated.

**Impact Statement**

This study throws light on the epidemiological and clinical profile of HIV-TB co-infected patients and deciphers the impact of TB on the recovery of CD4 counts in HIV positives.

## Introduction

Human immunodeficiency virus (HIV) and tuberculosis (TB) are the two most challenging infections faced by humanity. TB was almost on the verge of elimination in the developed countries till the late 1980s, when new cases of TB in HIV-positive patients and multidrug-resistant TB emerged and led to a resurgence of the disease [1].

As per the World Health Organization’s (WHO) latest estimates, the global incidence of HIV in the year 2021 was 1.5 million (1.1–2.0 million), and 650,000 (510,000–860,000) people died because of causes related to HIV [2]. In contrast, the estimated incidence of TB in the same year was 10.6 million, and 1.6 million patients died of the disease [3]. In brief, ~187,000 patients among TB deaths were also HIV positive [3]. Looking at these estimates, it can be said that HIV-TB co-infection increases the risk of mortality as compared to the risk with either disease alone. Estimates from India showed that the incidence of TB among HIV positives was 53,000 (range 36,000–72,000), amounting to 3.8% (range 2.6–5.2%), and among such HIV-TB co-infected patients, mortality was estimated to be 11,000 per lakh population (range 9,800–12,000), amounting to 0.78% (range 0.71–0.84%) [4].

Both HIV and TB infections impact each other’s progression. HIV disease is manifested by immunodeficiency that disrupts immune surveillance mechanisms, thus allowing for the development of opportunistic infections, among which TB is the most prevalent [1]. Even with effective immune reconstitution and the regain of high counts of clusters of differentiation 4 (CD4) cells with antiretroviral therapy (ART), HIV-positive patients have a 16 times (uncertainty interval 14–18) higher risk of developing TB during the course of the disease as compared to the background risk in the general population [5]. Likewise, TB also enhances the progression of HIV infection to AIDS and is a major cause of mortality in HIV-positive patients [6].

In developing countries, the possible reasons leading to the development of HIV-TB co-infection include factors like social stigma, culture, lack of awareness and inadequate availability of proper diagnostic and treatment facilities; such factors hinder the patients from getting diagnosed and treated [7]. Though India is one of the countries that bear the brunt of the HIV-TB co-infection burden, there is a paucity of data on the prevalence and risk factors of HIV-TB co-infection [8,10], with no data being available from the northern region of India to the best of our knowledge.

HIV-positive people, when treated with ART, usually result in a decrease in viral load and an increase in CD4+ T cell counts [11]. However, little is known about the recovery of these cells among HIV-TB co-infected patients, especially in the Indian population. This study was therefore done to understand the epidemiological and clinical profile of HIV-TB co-infected patients in northern India and to study the impact of TB on the recovery of CD4 counts.

## Methods

This was a hospital-based observational study conducted in the Department of Microbiology and ART centre under the Department of Medicine at King George’s Medical University, Lucknow, between May 2021 and June 2022. At the ART centre, HIV diagnosis is conducted in accordance with Strategy III of the National AIDS Control Organization (NACO), India, which involves a three-test protocol for all individuals suspected of infection [12]. During the study period, all the newly diagnosed HIV-positive patients were clinically evaluated for pulmonary (PTB) and extra-pulmonary TB (EPTB) and underwent radiological, histopathological and/or microbiological investigations for TB when indicated. Microbiological diagnosis was performed using a cartridge-based nucleic acid amplification test (CBNAAT) (Xpert MTB/RIF, Cepheid, USA). Patients diagnosed with HIV-TB co-infection were initiated on ART followed by anti-tubercular treatment (ATT) in accordance with the NACO guidelines [12].

During the study period, a total of 573 patients were newly diagnosed with HIV infection and enrolled at the ART centre. Among them, 80 (13.9%) patients were also diagnosed with TB within 2 to 3 weeks of enrolment. These 80 HIV-TB co-infected patients were evaluated for various demographic parameters, including age, gender, residential address, socio-economic status [assessed using the revised Brahm Govind (BG) Prasad classification to accommodate both rural and urban populations], occupation and marital status. Additionally, known risk factors for TB, including smoking, alcohol consumption and history of contact with a known case of TB, were also analysed [8,10]. Descriptive analysis of the variables was done using InStat Prism software version (9.4.1).

To study the impact of TB on CD4 count recovery, baseline CD4 count measurements were conducted for HIV-TB co-infected patients at the time of their enrolment at the ART centre. However, baseline CD4 was available for 79 of 80 patients. One patient absconded during the initial visit without providing a blood sample for CD4 count testing; therefore, only socio-demographic information was recorded.

Follow-up CD4 counts, conducted 6 to 8 months after initiating ART and ATT, were available for only 54 patients of the 79 HIV-TB co-infected patients. Among the remaining 25 patients, 7 were transferred to other ART centres, 4 were lost to follow-up and 14 died during the follow-up period, resulting in unavailable data. These 54 patients were studied to see the impact of TB on CD4 count recovery. For comparison, 50 age- and gender-matched consecutive HIV-only patients were selected as controls from a larger pool of 493 individuals. These 50 patients were selected based on availability of both their baseline and follow-up CD4 counts and absence of any sign or symptom of TB during the study period, thus requiring no TB-related investigations. The change in CD4 counts from baseline to follow-up was calculated and compared between the two groups.

For statistical analysis, all data were entered into the Microsoft Excel database. Frequencies were described in percentages. Parametric and non-parametric variables were tested for significance using a paired t-test and Wilcoxon signed rank test, respectively. Comparison in the change in CD4 counts in the two groups was done using the Mann–Whitney U test, and *P* values<0.005 were considered statistically significant.

Ethical clearance for the study was obtained from the Institutional Ethics Committee (*Ref.code: II-PGTSC-IIA/P21*). A waiver of consent was obtained for controls, and written informed consent was obtained from all the patients.

## Results

In this study, 80 out of 573 HIV positives were diagnosed with TB co-infection, representing a frequency of 13.96%. For these 80 HIV-TB co-infected patients, the mean age was 36.80±11.27 (range: 11–70 years) and male to female ratio was 3.1:1. Majority of these patients belonged to the Awadh region of Uttar Pradesh (U.P.) (*n*=69; 86.3%), with most being married (77.5%) and literate (76.3%) at varying levels of education. A portion was employed in government or private service (16.3%), and 41.25% belonged to socio-economic class I, as per the revised BG Prasad classification. The presence of smoking and alcohol abuse was found in 41.25 and 42.50% of patients, respectively. Sexual transmission was observed as the most common mode (85%) of transmission of HIV (Table 1).

**Table 1.** Epidemiological profile of HIV-TB co-infected patients

Variable	Category	No. of patient (%)
Age	10–20 >20–50 >50	4 (5.00%) 64 (80.00%) 12 (15.00%)
Gender	Male Female	63 (78.75%) 17 (21.25%)
Marital status	Married Unmarried Widowed	62 (77.50%) 14 (17.50%) 4 (5.00%)
Educational status	Illiterate Literate Primary school Secondary school College	19 (23.75%) 61 (76.25%) 19 (31.14%) 21 (34.42%) 21 (34.42%)
Socio-economic status	Upper class Upper middle class Middle class Lower middle class Lower class	33 (41.25%) 30 (37.50%) 6 (7.50%) 10 (12.50%) 1 (1.25%)
Probable mode of transmission of HIV	Sexual Blood transfusion IV drug user Vertical Probable unsafe injection Unknown	68 (85.00%) 2 (2.50%) 1 (1.25%) 3 (3.75%) 5 (6.25%) 1 (1.25%)
Type of TB	PTB EPTB Miliary	42 (52.50%) 37 (46.25%) 1 (1.25%)
Method of diagnosis of TB	Microbiological Clinical	16 (20.00%) 64 (80.00%)
Risk factor for TB	Smoking Alcohol	33 (41.25%) 34 (42.50%)

EPTB, extrapulmonary tuberculosis; IV drug, intravenous drug; PTB, pulmonary tuberculosis.

PTB was observed in 52.5% of cases, EPTB in 46.3% and miliary TB in 1.3% of cases. Of the 80 HIV-TB co-infected enrolled patients, CBNAAT was positive in only 16 (20%) patients. In remaining 64 patients (80%), TB was diagnosed clinically with additional support from histopathological findings in 1 (1.6%), radiological investigations in 41 (64.1%) or other pathological investigations in 11 (17.2%) cases. In the remaining 11 (17.2%) cases out of 64, the diagnosis of TB was based solely on WHO’s four-symptom screen (Table 1).

**Fig. 1.**
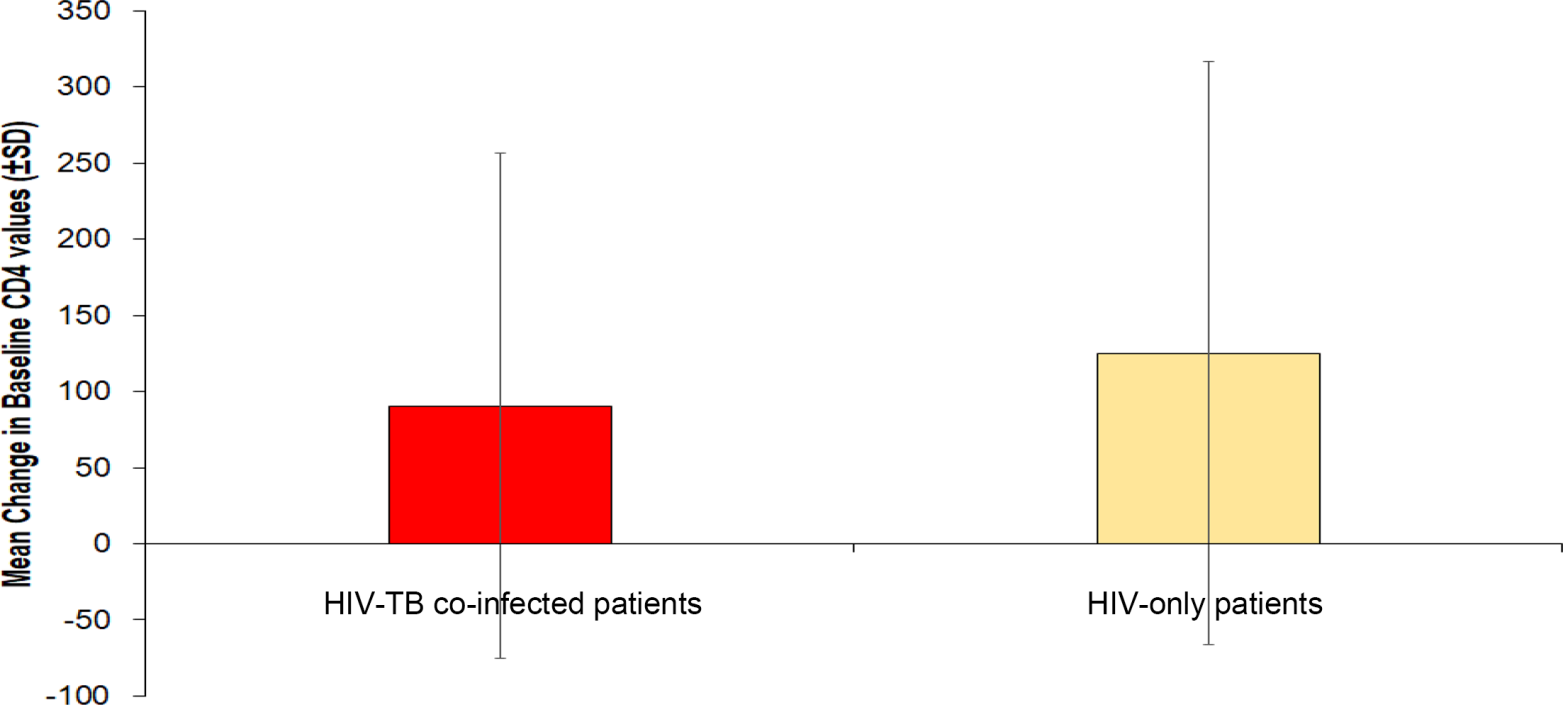
Comparison of change from baseline CD4 counts after 6–8 months of treatment in HIV-TB co-infected and HIV-only patients.

In the HIV-TB co-infected group, the mean baseline CD4 count was 194.52±162.27 cells per cubic millimeter (mm^−3^), and the mean follow-up (after receiving 6 to 8 months of ART and ATT) CD4 count was 285.09±170.33 cells mm^−3^. In HIV-only patients, the mean baseline CD4 count was 350.46±253.39 cells mm^−3^, which rose to the mean count of 475.72±283.40 cells mm^−3^ at follow-up. It was observed that patients with HIV-TB co-infection had significantly lower CD4 counts than the HIV-only patients both at the baseline (*χ*²=27.345; *P*<0.001) and at 6 months follow-up (*χ*²=14.994; *P*=0.001). When the change in CD4 counts was compared, it was observed that in both groups, the follow-up CD4 counts were significantly higher as compared to the baseline counts (the corresponding *Z* values were 2.858 and 2.132, respectively) (Table 2). However, the increase in counts was significantly higher (*Z* score: −2.01; *P*=0.04) in the HIV-only patients (increment in mean CD4 levels=125.26±191.48 cells mm^−3^, *t*=4.626, *P* value<0.001) than that in HIV-TB co-infected patients (increment in mean CD4 levels=90.57±165.60 cells mm^−3^, *t*=4.019, *P* value<0.001) (Fig. 1).

**Table 2.** Values of CD4 counts at baseline and at follow-up in HIV-TB co-infected patients and HIV-only patients enrolled in the study

	HIV-TB co-infected patients (*n*=54)		HIV-only patients (*n*=50)	
Value of CD4 count	At baseline	At follow-up	At baseline	At follow-up
<200 cells mm^−3^	32 (59.3%)	21 (38.9%)	12 (24.0%)	05 (10.0%)
200–499 cells mm^−3^	19 (35.2%)	27 (50.0%)	24 (48.0%)	28 (56.0%)
500 cells mm^−3^ and above	3 (5.6%)	6 (11.1%)	14 (28.0%)	17 (34.0%)
Wilcoxon signed rank test for change in CD4 levels	*Z*=2.858; *P*=0.004		*Z*=2.132; *P*=0.033	

## Discussion

India bears a huge burden of both HIV and TB infections, but data on the frequency of co-infections is limited. The present study was done to assess the frequency of HIV-TB co-infection among the HIV positives in north India, to study the epidemiological and clinical factors related to the co-infections and the impact of TB infection on HIV disease progression in terms of CD4 counts. Since both infections impact each other’s progression, the findings of this study become imperative for policymakers and other stakeholders.

HIV infection increases the risk of TB by causing an impaired immune response of the body to mycobacterial infection [13]. In the present study, the frequency of TB co-infection was found to be 13.96% among the HIV positives. Previous studies have reported the prevalence ranging from 9% in South Sudan to 42.1% in Tamil Nadu [14,15]. A recent meta-analysis showed that there was a huge geographical variation in the prevalence of HIV-TB co-infection, being 31.25% in African countries, 17.21% in Asian countries, 20.11% in European countries, 25.06% in Latin American countries and 14.84% in the USA. Possible explanations for such difference include factors such as: (1) prevalence of HIV in the general population, (2) variation in the availability of health services in different geographical areas, (3) level of awareness present among the general population of that area regarding utilization of health services, (4) time of start of ART during the course of disease and patient’s adherence to the prescribed ART and (5) sensitivity and specificity of diagnostic methods used in various studies. In the present study, in most HIV-infected individuals, TB was diagnosed based on clinical and radiological parameters only. Microbiological confirmation could be done for only 20% of HIV-TB co-infected patients based on CBNAAT. The WHO has endorsed CBNAAT as the upfront test for diagnosis of TB in HIV patients, both for PTB and EPTB, because of its high sensitivity, rapidity of diagnosis and ability to simultaneously detect rifampicin resistance [16]. Therefore, in the present study, CBNAAT was done on all the available samples. But the availability of adequate amounts of samples, particularly in EPTB, is a major limitation. The paucibacillary nature of the disease further compounds the problem, and hence, microbiological detection of TB in this population is often not possible. Lipoarabinomannan detection has been recommended in HIV-infected individuals for diagnosing TB [17]. The limitation of the present study was that it could not be done owing to limited resources.

Among the HIV-TB co-infected patients enrolled in this study, it was found that the combined frequency of EPTB (46.25%) and miliary TB (1.25%) was similar to that of PTB (52.5%). In contrast, it is known that among HIV-negative individuals, EPTB accounts for only 15–20% of all incident TB cases [12]. As per the NACO guidelines 2021, people living with HIV have unusual presentations of TB, including extra-pulmonary dissemination to several organs, as compared to the HIV-negative population due to deficient cell-mediated immunity. Though even in this population, PTB is still the most common form of active TB [12]. However, contrasting results with higher frequency of EPTB (58.1%) than PTB were found in another Indian study conducted by Wanchu *et al.* [18]. A systematic review by Shivakoti *et al.* showed that while many individual studies had shown increased odds of EPTB compared with PTB among the HIV-positive individuals, the true risk could not be assessed because of high heterogeneity between these studies [19]. Therefore, a high index of suspicion for both PTB and EPTB is imperative in this population.

In the present study, the majority of HIV-TB co-infected patients were males (78.75%). This finding corresponds with studies by Gautam *et al.*, Kamath *et al.*, Carvalho *et al.* and Oliveira *et al.*, where HIV-TB co-infected males were in a majority, the values being 81, 75.3, 53.2 and 75.4%, respectively [8,9, 20, 21]. Possible explanations are as follows: (1) in developing countries, it is expected that males are more exposed to mycobacteria as compared to females because of the socio-cultural dynamics and greater movement in the society, (2) higher burden of HIV and TB occurs individually among males more than females in the general population [22,23] and (3) there are still orthodox pockets in the country where males are preferably taken to a health care centre. Therefore, the true burden of the diseases is actually unknown among females, as indicated by the contradictory results found in a study by Mekonnen *et al.*, where the percentage of HIV-TB co-infection among women was relatively higher [24]. Population based studies are required to understand the true demographics of the disease.

In the present study, the majority of the HIV-TB co-infected patients belonged to the age group of 21–30 years (31.25%), followed by 31–40 years (28.75%). This finding corroborates the findings of a study conducted by Alemu *et al.* in which 55.6% of study participants were in the age group of 18–30 years [25]. Similar results were found in a study by Sube *et al.*, where the majority (39.3%) of patients with HIV-TB co-infection were aged between 25 and 34 years [14]. In studies conducted by Gautam *et al.* and Kamath *et al.* majority of study participants were in the age group of 30–45 years (71.3%) and 31–45 years (61.34%), respectively [8,9]. This may probably be explained by the higher prevalence of HIV in the sexually active age group. However, in a study conducted by Adhikari *et al.*, the maximum cases (12.5%) of HIV-TB co-infected patients were aged less than 20 years [26]. These variations may again be due to the hospital based nature of all the studies.

Most (77.5%) individuals among the HIV-TB co-infected patients were married. Our results were concordant with previous studies where the percentages of married individuals varied from 52.3% [27] to 62.2% [10]. The possible reason for this observation could be higher rates of sexual promiscuity.

Co-infection with TB was observed more frequently among literate HIV positives and in those belonging to upper class I (41.25%), followed by upper middle class II (37.5%). Similar results were found in a study by Aychiluhm *et al.* in which 55.1% of co-infected cases were literate [28]. The possible reason for this observation could be that the educated people with high socio-economic status are better aware and had an attitude of seeking the available healthcare facilities. However, conflicting results have been obtained in previous studies by Ahmad *et al.*, Gautam *et al.*, Fite *et al.* and Pondei *et al.*, where a majority of the co-infected individuals belonged to the low socio-economic status or were illiterate [6,8, 27, 29]. The authors postulated that this may be due to the relatively better quality of life among the literate and economically stable sector of society. Larger population-based studies are required to know the association of literacy and socio-economic status with HIV-TB co-infection.

In the present study, smoking and alcohol abuse were found in 41.25 and 42.50% of HIV-TB co-infected patients, respectively. Parrikar *et al.* found similar results with 54 and 19.9% of co-infected patients being alcohol consumers and smokers, respectively [10]. There are high chances of HIV treatment default among alcoholics, thereby increasing the risk of TB infection in them and smoking is an independent known risk factor for TB infection.

Baseline CD4 values of HIV-TB co-infected patients were significantly lower (*χ*^2^=27.266; *P*<0.001) than those of HIV-only patients. A meta-analysis from Ethiopia also found a significant association among low CD4 counts, advanced HIV stage as per WHO guidelines and HIV-TB co-infection [7]. TB infection may be the cause or effect of lower CD4 counts. Higher immunosuppression in people living with HIV possibly leads to increased vulnerability to TB infection, or co-infection with TB leads to further decrease in immunity and CD4 counts in HIV positives. Hence, intensifying surveillance of TB among HIV positives and of HIV among TB patients is urgently required.

When the follow-up CD4 counts were compared with the baseline values, a statistically significant increase was observed in both the groups, HIV-TB co-infected patients and HIV-only patients. This phenomenon has also been observed in previous studies [8,9]. But when the two groups were compared, the increment in HIV-only patients was found to be statistically higher than that in HIV-TB co-infected patients. A study done on the kinetics of CD4 count among the two groups showed that at 5 years after the initiation of ART, the median CD4 counts were significantly lower in HIV-TB co-infected patients. They also concluded that TB slows the rate of recovery of CD4 counts during the first 6 months after ART initiation [30]. Further prospective studies with a larger sample size should be conducted for a better understanding of the CD4 count profile among the HIV-only and HIV-TB co-infected population.

## Conclusion

To conclude, the main findings of this study are a frequency of 13.96% of HIV-TB co-infection among HIV positives, co-infection being more common among younger age group, in males, literate people and those belonging to higher socio-economic status. Also, a significant rise in CD4 counts was observed in both the groups receiving ART, but the rise was significantly higher among the HIV-only group as compared to the HIV-TB co-infection group. Therefore, further prospective studies with a larger sample size are needed for a better understanding of the impact of TB on CD4 count profile.

## Abbreviations

ART: antiretroviral therapy
ATT: anti-tubercular treatment
BG: Brahm Govind
CD4: clusters of differentiation 4
EPTB: extra-pulmonary TB
HIV: human immunodeficiency virus
NACO: National AIDS Control Organization
PTB: pulmonary TB
TB: tuberculosis
WHO: World Health Organization
